# A comparative study of unpasteurized and pasteurized frozen whole hen eggs using size-exclusion chromatography and small-angle X-ray scattering

**DOI:** 10.1038/s41598-022-12885-z

**Published:** 2022-06-02

**Authors:** Yoshiki Oka, Hiroko Yukawa, Hisashi Kudo, Koji Ooka, Manami Wada, Shunji Suetaka, Mari Chang, Hidenobu Kawai, Ryouji Tanaka, Masahiro Ichikawa, Takahisa Suzuki, Yuuki Hayashi, Akihiro Handa, Munehito Arai

**Affiliations:** 1 Department of Life Sciences, Graduate School of Arts and Sciences, The University of Tokyo, 3-8-1 Komaba, Meguro, Tokyo 153-8902 Japan; 2 R&D Division, Institute of Technology Solution, Kewpie Corporation, Sengawa Kewport, 2-5-7 Sengawa, Chofu, Tokyo 182-0002 Japan; 3 Department of Physics, Graduate School of Science, The University of Tokyo, 3-8-1 Komaba, Meguro, Tokyo 153-8902 Japan; 4 Environmental Science Center, The University of Tokyo, 7-3-1 Hongo, Bunkyo, Tokyo 113-0033 Japan; 5 Division of Life Science, School of Science and Engineering, Tokyo Denki University, Ishizaka, Hatoyama-machi, Hiki-gun, Saitama 350-0394 Japan; 6 Present Address: Graduate School of Science, Technology and Innovation, Kobe University, 1-1 Rokkodai-cho, Nada, Kobe, Hyogo 657-8501 Japan; 7 Present Address: Komaba Organization for Educational Excellence, College of Arts and Sciences, The University of Tokyo, 3-8-1 Komaba, Meguro, Tokyo 153-8902 Japan

**Keywords:** Molecular conformation, SAXS, Protein aggregation

## Abstract

Hen eggs are rich in proteins and are an important source of protein for humans. Pasteurized frozen whole hen eggs are widely used in cooking and confectionery and can be stored for long periods. However, processed eggs differ from raw eggs in properties such as viscosity, foaming ability, and thermal aggregation. To develop pasteurized frozen whole egg products with properties similar to those of unpasteurized whole eggs, it is necessary to establish a method that can differentiate between the two egg types with respect to the structures of their proteins. In this study, size-exclusion chromatography (SEC) and SEC coupled with small-angle X-ray scattering (SEC-SAXS) were successfully used to differentiate between the proteins in unpasteurized and pasteurized frozen whole eggs. We found that proteins in the plasma fraction of egg yolk, especially apovitellenins I and II, formed large aggregates in the pasteurized eggs, indicating that their structures are sensitive to temperature changes during pasteurization, freezing, and thawing. The results suggest that SEC and SEC-SAXS can be used to differentiate between unpasteurized and pasteurized frozen whole eggs. Additionally, they may be useful in determining molecular sizes and shapes of multiple components in various complex biological systems such as whole eggs.

## Introduction

Hen eggs are rich in proteins and are an important source of protein for humans^1^. Processed hen eggs that have been pasteurized and frozen can be stored for long periods and are widely used in cooking and the production of confectionery^2^. Pasteurization, typically at ~ 60 °C, is performed to kill *Salmonella* spp., *Escherichia coli*, and other bacteria in eggs, as high-temperature sterilization causes thermal denaturation of egg proteins^2^. The process is usually followed by rapid freezing to produce pasteurized frozen whole hen egg products. The frozen products are thawed before used in cooking. However, temperature changes owing to pasteurization, freezing, and thawing can cause changes in the properties of hen eggs. For example, these changes can result in a decrease in the foaming ability of eggs when making sponge cakes as well as a decrease in heat aggregation when making egg soup^3–5^. Therefore, the development of pasteurized frozen whole egg products with properties similar to those of unpasteurized whole eggs has been an important issue in food science and industry.

A hen egg consists of a shell, egg white, and yolk. The egg white and yolk contain several proteins with diverse structures^6^. Approximately half of the protein content of egg white is ovalbumin^7^. Other proteins in egg white include ovotransferrin (conalbumin), ovomucoid, lysozyme, and ovomucin^7^. In contrast, egg yolk is rich in lipoproteins, which are lipid-protein complexes. Egg yolk consists of a soluble fraction (called plasma) and an insoluble fraction (called granules) containing large particles^7^. The main components of the plasma fraction are low-density lipoproteins (LDLs), very-low-density lipoproteins (VLDLs), and soluble proteins (mainly livetin), while the main component of the granule fraction is high-density lipoproteins (HDLs)^8^. Temperature changes during pasteurization, freezing, and thawing may induce structural changes in these proteins by altering their molecular size and shape^7,9,10^. As a result, the viscosity, foaming ability, and heat aggregation of pasteurized frozen whole eggs may be different from those of unpasteurized whole eggs^11,12^. Therefore, elucidating the differences between unpasteurized and pasteurized frozen whole eggs, with respect to the proteins they contain, at the molecular level would be useful in the development of pasteurized frozen whole egg products with foaming properties similar to those of unpasteurized whole eggs. Thus, it is necessary to establish a method that can be used to evaluate the molecular size and shape of proteins in these eggs.

Size-exclusion chromatography (SEC) is a high-performance liquid chromatographic (HPLC) technique that is useful for studying protein structure and aggregation. In SEC, proteins with large molecular weights and extended structures elute early, while proteins with small molecular weights and compact structures elute late. In many cases, the ultraviolet (UV) absorption of a protein sample eluted in SEC is measured to quantify the amount of the protein (called SEC-UV measurement). Furthermore, sodium dodecyl sulphate–polyacrylamide gel electrophoresis (SDS-PAGE) analysis can identify the components of the elution peaks. Recently, SEC combined with small-angle X-ray scattering (SAXS), which is referred to as the SEC-SAXS method, has attracted much attention^13–16^. In the SAXS technique, irradiation of a protein solution with X-rays produces scattering curves, which can be analyzed to obtain the size and shape of the protein molecules. Therefore, it is possible to use the SEC-SAXS method to determine the molecular sizes and shapes of multiple components in a complex system such as whole egg. This can be achieved by separating the components using SEC, followed by analysis of the individual components by SAXS.

In this study, we investigated the differences in the molecular sizes and shapes of proteins in unpasteurized and pasteurized frozen whole hen eggs using SEC-UV and SEC-SAXS. The results showed clear differences between the proteins in the eggs. In particular, proteins in egg yolk plasma, especially apovitellenins I and II, formed large aggregates in pasteurized frozen whole eggs. The results suggest that SEC-UV and SEC-SAXS can be effectively used to clarify the differences between unpasteurized and pasteurized frozen whole eggs. Additionally, these techniques may be useful in determining molecular sizes and shapes of multiple components in various complex biological systems such as whole eggs.

## Results

### SEC-UV analysis

To prevent clogging of the SEC column in HPLC experiments, samples are usually centrifuged and filtered through a 0.45 μm filter before they are introduced into the column. The granule fraction of egg yolk contains molecular species with diameters of 0.3–2.0 μm^17,18^. Therefore, some proteins in the granule fraction and large aggregates with diameters of 0.45 μm or greater were removed before sample analysis (Supplementary Fig. 1). In addition, since whole eggs, especially pasteurized frozen whole eggs, are highly viscous and difficult to analyze in HPLC experiments, all samples were diluted to 1/25 using the elution buffer (50 mM sodium phosphate [pH 7.8] and 550 mM NaCl). The pH of the buffer was adjusted to the pH of the raw whole eggs^19^. It is reported that ~ 550 mM NaCl dissociates the granules in egg yolk^20^. Thus, the viscosity of the pasteurized frozen whole eggs was reduced to allow for the HPLC experiments to be performed successfully.

**Figure 1:**
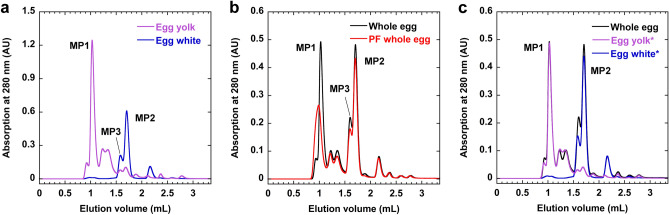
Elution profiles obtained from the SEC-UV analyses. The three major peaks are marked as MP1, MP2, and MP3. (**a**) Unpasteurized egg yolk and egg white. (**b**) Unpasteurized whole eggs and pasteurized frozen (PF) whole eggs. (**c**) The profiles of unpasteurized egg yolk and egg white superimposed on that of unpasteurized whole eggs after the intensities of the maximum peaks for egg yolk and egg white (MP1 and MP2, respectively) were matched to those of whole eggs. *means that the intensity of the profile was corrected. The figures were created with KaleidaGraph 4.1.0 (Synergy Software, Reading, PA, USA; https://www.synergy.com/).

First, unpasteurized egg yolk and egg white were subjected to SEC-UV measurements as control samples (Fig. 1a). Several peaks were observed in the elution volume range of 0.9–1.5 mL for unpasteurized egg yolk; however, there were almost no peaks after 1.5 mL of elution. In contrast, there were almost no peaks in the range of 0.9–1.5 mL for unpasteurized egg white; however, large peaks were observed after 1.5 mL.

Next, unpasteurized and pasteurized frozen whole eggs were subjected to the SEC-UV analysis (Fig. 1b). Overall, three major peaks and several smaller peaks were observed. The major peaks were named MP1, MP2, and MP3 in descending order of height. The elution volumes of MP1, MP2, and MP3 were 0.9–1.2 mL, 1.65–1.8 mL, and 1.5–1.65 mL, respectively. In the range of 0.9–1.5 mL, the elution profile for the unpasteurized whole eggs was similar to that for the unpasteurized egg yolk, and when normalized by the height of the maximum peak, the shape of the profile for the unpasteurized whole eggs was in good agreement with that of the yolk (Fig. 1c). Similarly, after 1.5 mL, the elution profile for the unpasteurized whole eggs was in good agreement with that for the unpasteurized egg white when normalized by the height of the maximum peak (Fig. 1c). Therefore, elution peaks in the range of 0.9–1.5 mL mainly represented egg yolk components for the whole egg sample, whereas peaks after 1.5 mL were mainly for egg white components.

The elution profile around MP2 and MP3 for the pasteurized frozen whole eggs was similar to that for the unpasteurized whole eggs. However, the MP1 of the pasteurized eggs eluted earlier and had a lower intensity compared to the corresponding peak of the unpasteurized eggs, suggesting the presence of large molecular species (Fig. 1b). The low intensity of MP1 for the pasteurized eggs was probably because some lipoproteins formed large aggregates during the heating, freezing, and thawing processes and were removed during the centrifugation and filtration stages before the HPLC analysis.

### SDS-PAGE

**Figure 2:**
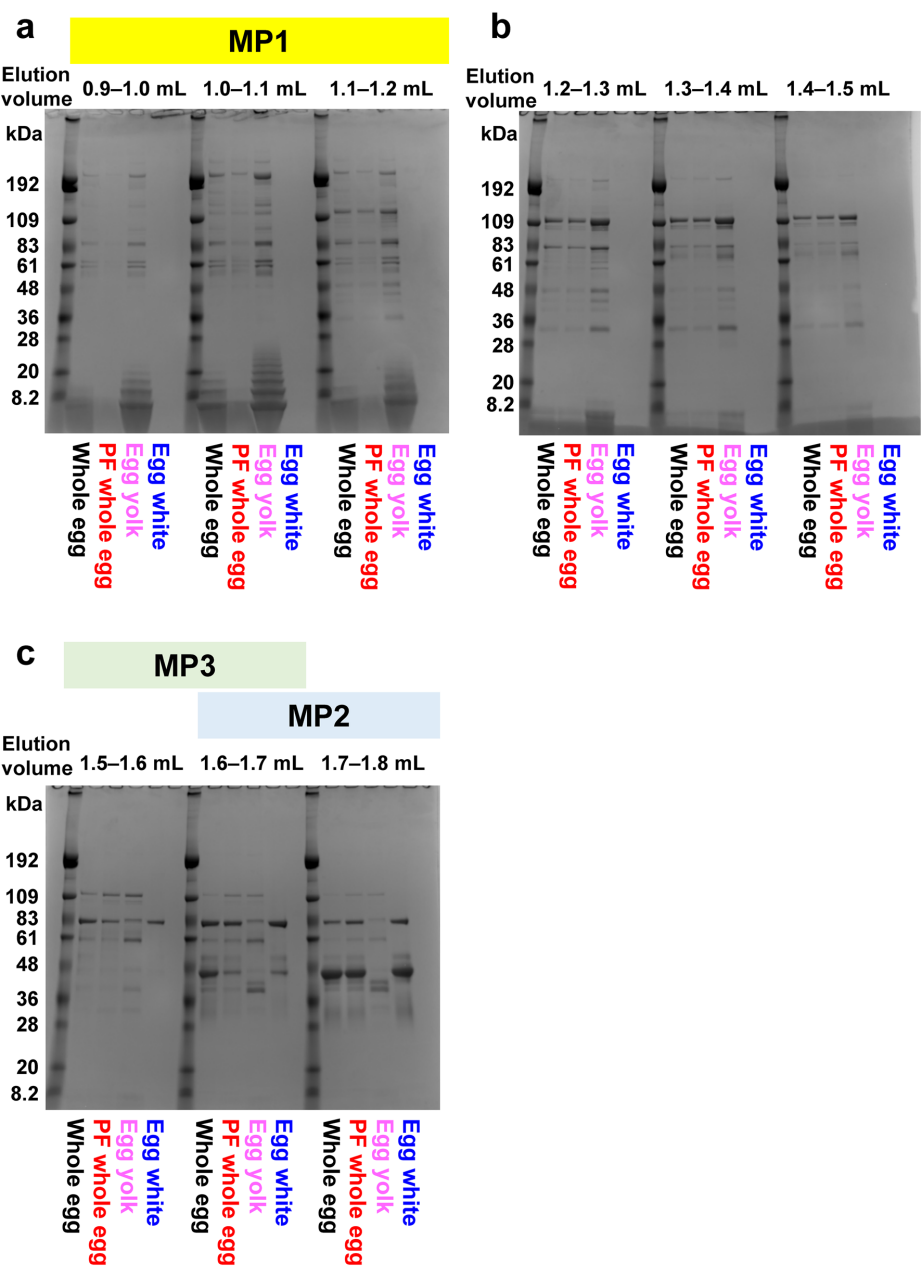
SDS-PAGE results for SEC elution fractions. For each fraction, the lanes for molecular weight markers, unpasteurized whole eggs, pasteurized frozen (PF) whole eggs, unpasteurized egg yolk, and unpasteurized egg white are shown. (**a**) Fractions corresponding to MP1 (elution volume of 0.9–1.2 mL). (**b**) Fractions for the elution volume of 1.2–1.5 mL. (**c**) Fractions corresponding to MP2 and MP3 (elution volume of 1.5–1.8 mL).

The eluate from the SEC column was collected in fractions (0.1 mL each). SDS-PAGE was performed to evaluate the proteins in the fractions (Fig. 2, Supplementary Fig. 2). For unpasteurized egg yolk, bands around the 192, 109, 83, 61, 36, 20, and 8.2 kDa markers were found in the 0.9–1.2 mL fraction corresponding to MP1 (Fig. 2a). Intense bands near the 192, 83, 20, and 8.2 kDa markers were assigned to apovitellenin VI (203 kDa) and γ-livetin (203 kDa), apovitellenin V (85 kDa), apovitellenin II (20 kDa) and apovitellenin I dimer (17 kDa), and apovitellenin I monomer (9 kDa) and apolipoprotein C II (5 kDa), respectively, based on previous studies (Supplementary Table 1)^8,21^. Apovitellenin I was considered to exist in both dimeric and monomeric forms, as reported previously^8^. These proteins are mainly LDLs in the plasma fraction of egg yolk, whereas apovitellenins I and II and apolipoprotein C II are components of VLDLs. Note that the proteins with low molecular weights that are eluted in the MP1 fraction should be contained in large complexes, rather than existing in isolation.

In the 1.2–1.5 mL fraction, an intense band was observed around the 109 kDa marker (Fig. 2b) that may correspond to apovitellin 3 + 4 (110 kDa), an HDL protein in the granule fraction of egg yolk. Thus, for unpasteurized egg yolk, components of the plasma and granule fractions were eluted separately.

A particularly intense band near the 48 kDa marker in the 1.6–1.8 mL fraction corresponding to MP2 was obtained for unpasteurized egg white (Fig. 2c). This band has been previously assigned to ovalbumin (45 kDa)^22^, which is abundant in egg white. In the 1.5–1.6 mL fraction corresponding to MP3, the band around the 83 kDa marker represents ovotransferrin (78–80 kDa)^22^. This band was found to be the most intense (Fig. 2c), which indicates that the major component of this fraction was ovotransferrin. Another fraction with an elution volume of 2.1–2.4 mL produced a band around the 8.2 kDa marker, which may correspond to lysozyme (14 kDa)^22^ (Supplementary Fig. 2). Although egg white contains ovomucin (250–700 kDa)^23^, no bands corresponding to molecular weights above 200 kDa were clearly detected (Supplementary Fig. 2). This was probably because high-molecular-weight components were removed from the samples before the HPLC analysis was performed. In addition, the band denoting ovomucoid (21 kDa)^24^ was not clearly observed.

The bands obtained for the egg yolk and egg white were compared to those obtained for the whole egg samples. The results showed that the bands for the whole egg samples were similar to those for the unpasteurized egg yolk in the 0.9–1.2 mL fraction corresponding to MP1 (Fig. 2a). However, some bands around the 20 kDa marker were almost completely lost for the pasteurized frozen whole eggs. According to the band assignments above, the bands that disappeared were those of apovitellenins I and II. Interestingly, these bands were not observed at any elution volume for the pasteurized frozen whole eggs (Supplementary Fig. 2). Therefore, apovitellenins I and II probably formed large aggregates and were removed before the HPLC analysis. In contrast, after 1.5 mL of elution, there was no significant difference in the SDS-PAGE bands for the egg white and whole egg samples.

In summary, the results of the SEC-UV and SDS-PAGE suggest that protein components that were affected by temperature changes during pasteurization, freezing, and thawing were mainly those eluted in 0.9–1.2 mL, which were the components of the egg yolk plasma. In addition, the results suggest that apovitellenins I and II form large aggregates in pasteurized frozen whole eggs.

### SEC-SAXS analysis

**Figure 3:**
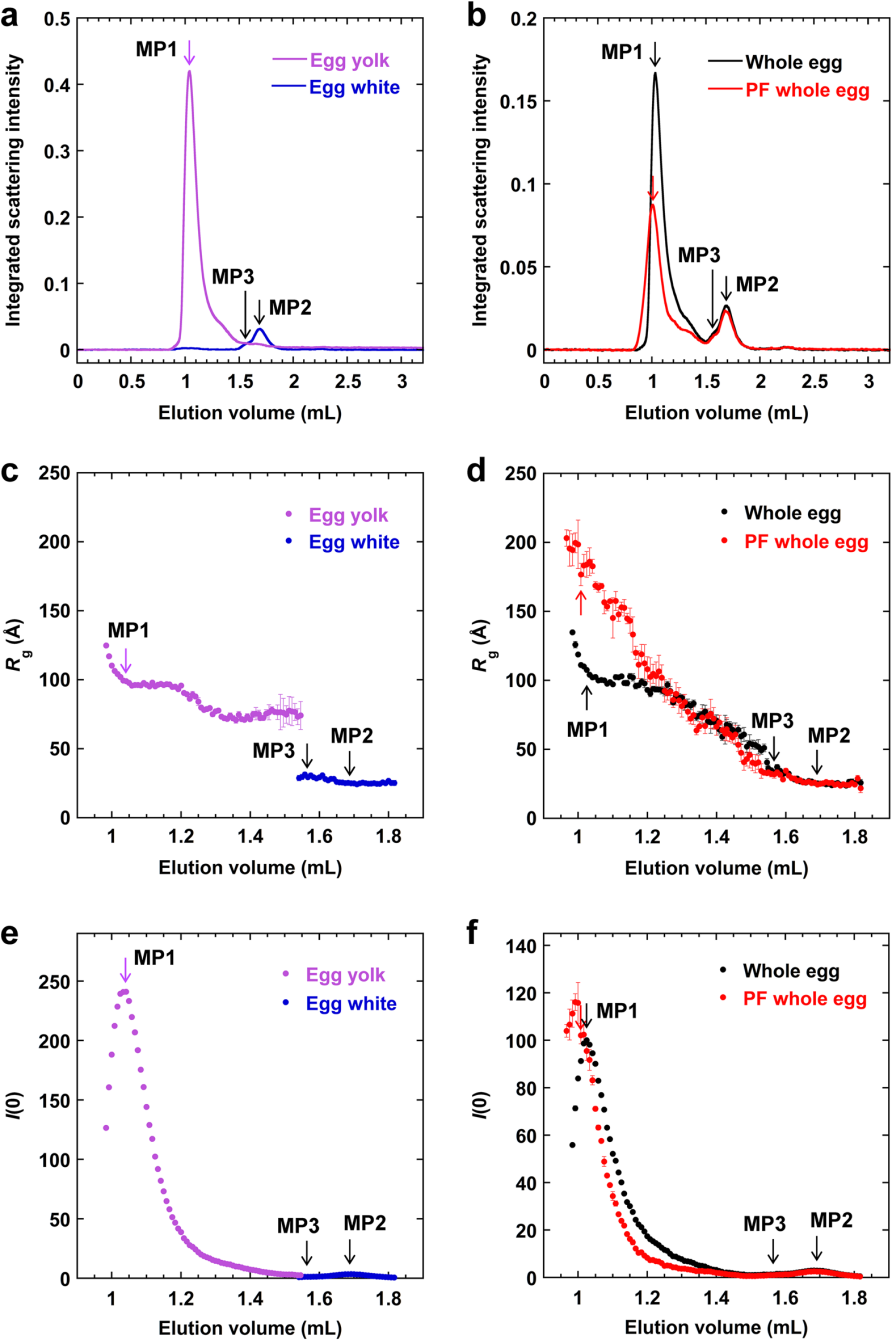
Elution profiles obtained from the SEC-SAXS analyses. The three major peaks are marked as MP1, MP2, and MP3. Elution profiles of (**a**) unpasteurized egg yolk and egg white and (**b**) unpasteurized and pasteurized frozen (PF) whole eggs as monitored based on their integrated scattering intensities. (**c,d**) *R*_g_ and (**e,f**) *I*(0) values determined from the Guinier plots for (**c,e**) unpasteurized egg yolk and egg white and (**d,f**) unpasteurized and pasteurized frozen whole eggs. The arrows show the positions of the major peaks, at which SAXS data analyses shown in Figs. 4 and 5 were performed. The error bars indicate fitting errors. The integrated scattering intensity and *I*(0) values are in arbitrary units. The figures were created with KaleidaGraph 4.1.0 (Synergy Software, Reading, PA, USA; https://www.synergy.com/).

The next experiment we performed was analysis of the egg samples using the SEC-SAXS technique^13–16^. In this experiment, the eluate from the SEC column attached to the HPLC system was irradiated with X-rays, and the X-ray scattering images were continuously analyzed (Supplementary Fig. 3). A series of 390 scattering curves (frames) were obtained for each sample. For each scattering curve, an integrated scattering intensity (i.e., the sum of the scattering intensities, *I*(*Q*), at all scattering vectors, *Q*) was calculated and plotted against the elution volume to obtain the elution profile (Fig. 3a,b). Similar to the SEC-UV results, the elution profile of egg samples monitored by their integrated scattering intensities showed two major peaks corresponding to MP1 and MP2. In addition, the peak corresponding to MP3 was present as a shoulder on the left side of the MP2. The resolution of the elution profiles that were monitored via SAXS were lower compared to those monitored via UV absorption, which may be attributed to the exposure time of 10 s for measuring a single scattering curve. The peak intensities of MP1 and MP2 were comparable in the SEC-UV analysis; however, the integrated scattering intensity of MP1, which corresponds to the protein that eluted first, was significantly larger than that of MP2 in the SEC-SAXS experiment. It is reported that integrated scattering intensities increase if samples have high molecular weights and/or are tested at high concentrations. Moreover, because MP1 contains components with high molecular weights (see Fig. 2), the integrated scattering intensity of MP1 was higher than that of MP2.

**Figure 4:**
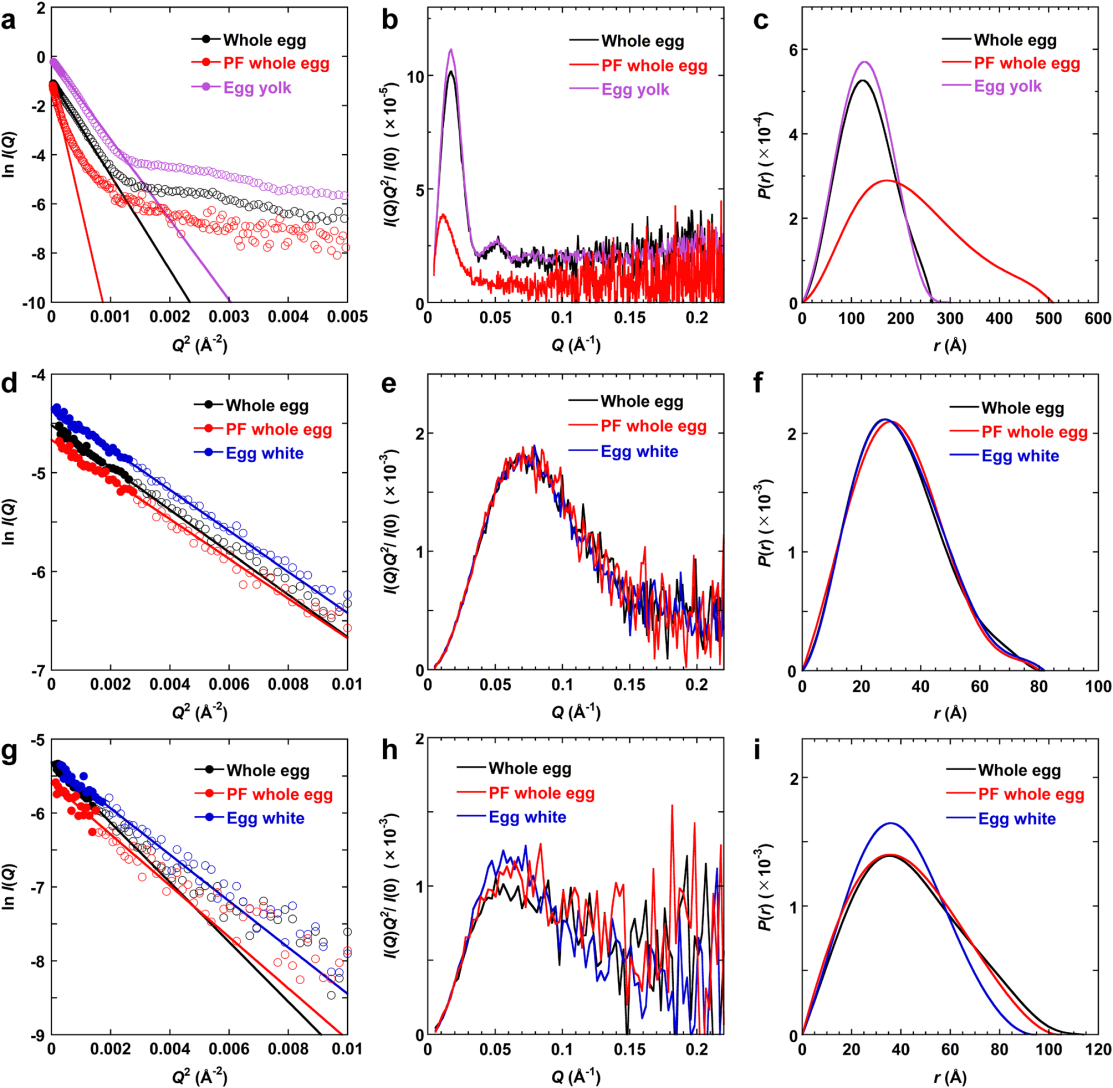
SAXS analysis of egg samples. The SAXS data at the tops of MP1 (**a–c**), MP2 (**d–f**), and MP3 (**g–i**). (**a,d,g**) Guinier plots. Data points used for fitting into Eq. (1) based on Guinier approximation are shown by filled circles. The continuous lines were obtained from the fitting. The *I*(*Q*) values are in arbitrary units. (**b,e,h**) Kratky plots. (**c,f,i**) *P*(*r*) functions. For the Kratky plots and *P*(*r*) functions, the curves divided by *I*(0) are shown. The figures were created with KaleidaGraph 4.1.0 (Synergy Software, Reading, PA, USA; https://www.synergy.com/).

The Guinier plot (i.e., ln *I*(*Q*) vs. *Q*^2^) of a scattering curve can be analyzed in order to estimate radius of gyration, *R*_g_, and zero-angle scattering intensity, *I*(0), based on the Guinier approximation^25,26^ (see “Methods” section, Fig. 4a). *R*_g_ is a measure of molecular size, whereas *I*(0) is a parameter that is proportional to the molecular weight and concentration of a sample. The *R*_g_ and *I*(0) values were determined at elution volumes for which integrated scattering intensities were high (Fig. 3c–f). The values at the tops of MP1, MP2, and MP3 are shown in Table 1. In other regions, low scattering intensities prevented accurate determination of these values. Detailed analyses of the sizes and shapes of protein components in the MP1, MP2, and MP3 regions were performed and the results are presented below.

**Table 1:** Radius of gyration (*R*_g_) and zero-angle scattering intensity (*I*(0)) at the three major peaks (MP1–3) obtained from the SEC-SAXS analyses.

Sample	MP1			MP2			MP3			*I*(0) ratio (MP1/MP2)^3^
	Frame #^1^	*R*_g_ (Å)	*I*(0)^2^	Frame #	*R*_g_ (Å)	*I*(0)	Frame #	*R*_g_ (Å)	*I*(0)	
Whole egg	122	108 ± 1^4^	100 ± 1	202	25.4 ± 0.5	2.90 ± 0.03	186	35 ± 2	1.30 ± 0.04	34.5
PF whole egg^5^	120	177 ± 8	102 ± 3	202	24.6 ± 0.5	2.50 ± 0.03	186	32 ± 2	0.99 ± 0.04	40.8
Egg yolk	124	99.0 ± 0.6	241 ± 1	–	–^6^	–	–	–	–	–
Egg white	–	–	–	202	25.0 ± 0.3	3.45 ± 0.03	186	31 ± 2	1.32 ± 0.04	–

^1^The number of the scattering curve used for the analysis among a series of 390 curves in the SEC-SAXS measurement. These positions in the elution profiles are shown by arrows in Fig. 3.

^2^*I*(0) values were normalized to 100 based on the *I*(0) at the MP1 of unpasteurized whole eggs.

^3^Ratio of the *I*(0) value at MP1 to that at MP2.

^4^Fitting errors to Eq. (1).

^5^Pasteurized frozen whole egg.

^6^Not detected.

### MP1 (egg yolk plasma)

The MP1 fraction contains multiple proteins mainly from egg yolk plasma, as described above (Fig. 2a). The scattering intensity of such a polydisperse system is obtained as the summation of the scattering intensities of the individual components, weighted by their respective volume fractions^27^. Let us consider two typical cases. Suppose first that there is a mixture of components with large and small *R*_g_ values. Since the *I*(0) of a globular solute is proportional to the square of the solute volume^25^, the scattering from the large component is dominant in the very small-angle region, while the scattering from the small component is negligible. Hence, the *R*_g_ obtained by the Guinier approximation in the very small-angle region approximately corresponds to the *R*_g_ of the large component. Beyond the very small-angle region, the scattering from the large component sharply decreases^25^. Consequently, the scattering from the small component becomes dominant, and a second linear region may be observed in the Guinier plot. Thus, if there is a very large difference in the size of the coexisting components, their *R*_g_ values may be obtained from the Guinier approximation at different regions in the Guinier plot. For SEC-SAXS measurements, however, such analysis is unlikely to be applicable because components with different sizes are separated by SEC and only components with similar sizes should coexist in each fraction.

Second, suppose that there is a mixture of two components with similar sizes. By expanding the equation of the Guinier approximation, we obtain $I_{1} (Q)\sim I_{1} (0)[1 - R_{{\text{g,1}}}^{2} Q^{2} /3]$ and $I_{2} (Q)\sim I_{2} (0)[1 - R_{{\text{g,2}}}^{2} Q^{2} /3]$ for components 1 and 2, respectively, at small angles. Since the scattering intensity is additive^27^, the observed scattering intensity *I*_obs_(*Q*) ($\sim I_{{{\text{obs}}}} (0)[1 - R_{{\text{g,obs}}}^{2} Q^{2} /3]$) is equal to *I*_1_(*Q*) + *I*_2_(*Q*), and thus the square of the observed *R*_g,obs_ can be approximated by the linear combination of the *R*_g_^2^ values of the two components as $R_{{\text{g,obs}}}^{2} = \left[ {I_{{1}}^{{}} (0)R_{{\text{g,1}}}^{2} + I_{{2}}^{{}} (0)R_{{\text{g,2}}}^{2} } \right]/I_{{{\text{obs}}}} (0)$, where *I*_obs_(0) = *I*_1_(0) + *I*_2_(0)^28^. A similar equation holds for the case of more than two components. Such analysis has been successfully applied in previous studies to track changes in molecular size associated with equilibrium unfolding transitions and kinetic folding reactions of proteins^28–30^. Therefore, even for a polydisperse system, the observed *R*_g_ in SEC-SAXS measurements is an indicator of an average molecular size. Similarly, the maximum length, *D*_max_, and modeled structure are considered to represent the averaged size and shape of similar components eluted at the same time.

The Guinier plots at the top of MP1 showed that the *R*_g_ value obtained for pasteurized frozen whole eggs was the highest, followed by that for unpasteurized whole eggs and egg yolk (Fig. 3c,d, Table 1); this was consistent with the elution order in the SEC (Figs. 1, 3a,b). The results indicate that the aggregates in the pasteurized frozen whole eggs were larger than those in the unpasteurized whole eggs. Similarly, the *I*(0) value for pasteurized frozen whole eggs was higher than that for unpasteurized whole eggs (Fig. 3f), although the SEC-UV data showed a lower concentration of MP1 components in the pasteurized eggs than in the unpasteurized eggs. Since *I*(0) is proportional to both the molecular weight and concentration of a sample, the results again indicate that larger aggregates were formed in the pasteurized frozen whole eggs. The MP1 component of the unpasteurized whole eggs, which was mainly from the egg yolk fraction, had a slightly larger *R*_g_ value than the MP1 component of the unpasteurized egg yolk sample had (Table 1). This suggests that egg yolk and egg white interact to form a complex in whole eggs.

Figure 4b shows the Kratky plots (i.e., *I*(*Q*)*Q*^2^ vs. *Q*) of the scattering curves at the top of MP1. It is reported that a peak in a Kratky plot indicates that a solute has a globular structure^26,31^, whereas the absence of a peak indicates that a solute has an extended structure. A peak was found in the Kratky plots for both the unpasteurized and pasteurized frozen whole eggs, indicating that the major large components in MP1 formed globular and compact structures. A scattering curve is in reciprocal space and its abscissa is in the unit of reciprocal distance; therefore, the position of a peak at a small angle indicates a large molecular size. The peak in the Kratky plot for the unpasteurized whole eggs was at *Q* = 0.015 Å^−1^, whereas that for the pasteurized frozen whole eggs was at *Q* = 0.010 Å^−1^. This again indicates that the average molecular size of the MP1 components in the pasteurized frozen whole eggs was larger than that in the unpasteurized sample.

The pairwise distance distribution function *P*(*r*) was obtained by calculating the inverse Fourier transform of the scattering curve (Fig. 4c). This function corresponds to the distribution of the distance between two atoms in a solute. The value of *r* at the peak of *P*(*r*) is close to *R*_g_, and a symmetrical distribution function implies a spherical structure. Furthermore, the point at which *P*(*r*) becomes zero is the maximum length, *D*_max_, which is the largest interatomic distance in a solute. Generally, if *D*_max_ is high and the distribution function is spread to the right side, it suggests that the solute has an extended structure. The results showed that unpasteurized whole eggs and egg yolk had almost symmetrical *P*(*r*) functions and a *D*_max_ of ~ 260 Å, while pasteurized frozen whole eggs had a *P*(*r*) spread to the right side and a *D*_max_ of ~ 500 Å (Fig. 4c). These results indicate that the MP1 components of the egg yolk plasma have, on average, a spherical structure when unpasteurized; however, it undergoes structural changes into approximately two-fold elongated structures as a result of pasteurization, freezing, and thawing.

**Figure 5:**
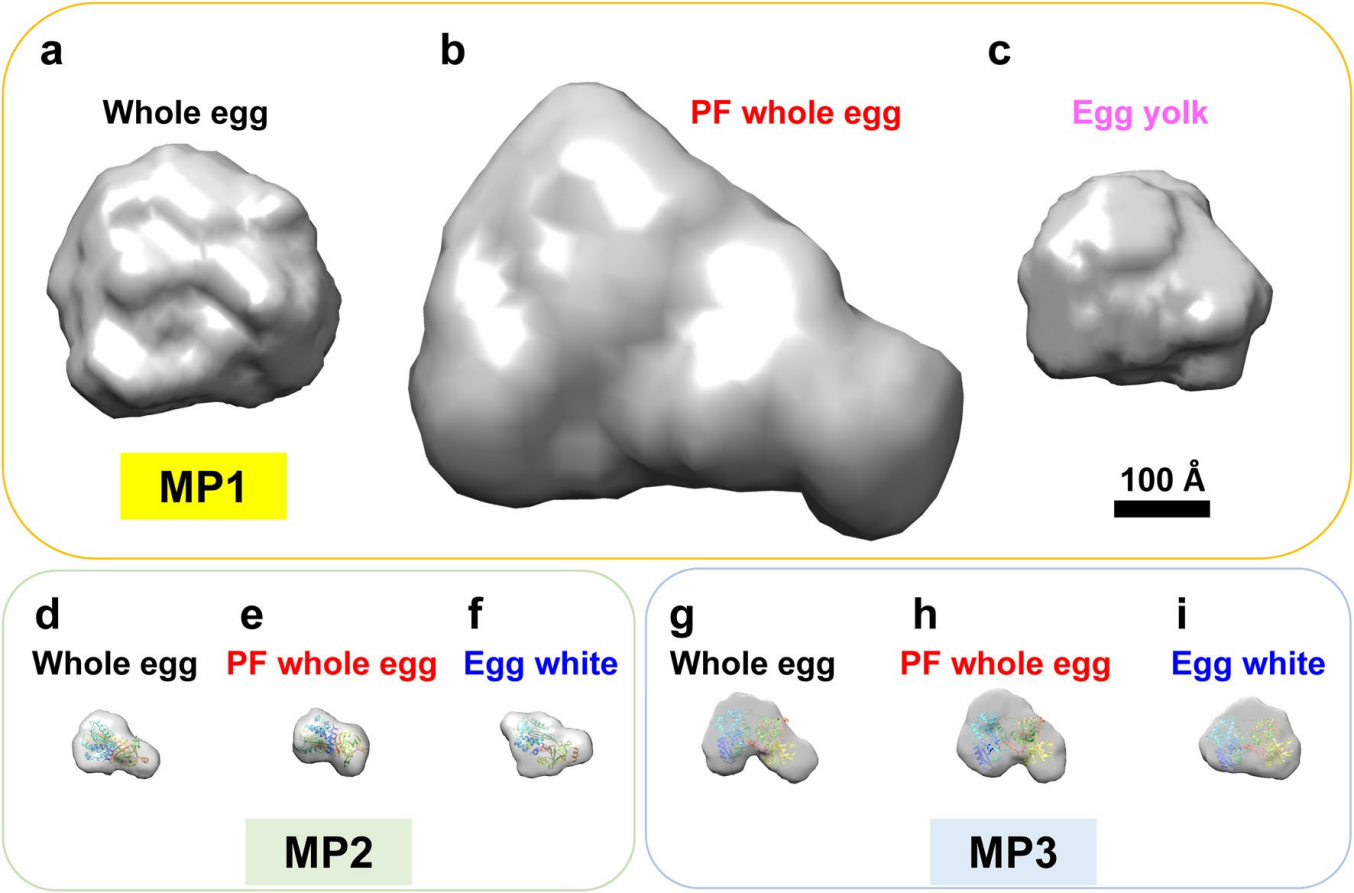
Structural modeling of egg samples using DAMMIF software. The size of each model shown is proportional to the *D*_max_. Model structures at the top of MP1 (**a–c**), MP2 (**d–f**), and MP3 (**g–i**) for (**a,d,g**) unpasteurized whole eggs, (**b,e,h**) pasteurized frozen (PF) whole eggs, (**c**) unpasteurized egg yolk, and (**f,i**) unpasteurized egg white. The crystal structures of ovalbumin and ovotransferrin are superimposed onto the model structures of MP2 and MP3, respectively. All structures are displayed on the same scale. A 100 Å scale bar is shown. The figures were drawn using Chimera software version 1.14 (Resource for Biocomputing, Visualization, and Informatics, University of California, San Francisco, San Francisco, CA, USA; https://www.cgl.ucsf.edu/chimera/)^53^.

Based on the SAXS data, the averaged overall structures of the MP1 components were modeled using DAMMIF^32^ and GASBOR softwares^33^ (Fig. 5, Supplementary Fig. 4). The *R*_g_ and *D*_max_ of the model structures (Table 2) were consistent with those obtained from the Guinier plot and *P*(*r*) function (Table 1), respectively, which indicates the validity of the structural modeling. Furthermore, the model structures for unpasteurized whole eggs and egg yolk were almost spherical (Fig. 5a,c), whereas the structure for pasteurized frozen whole eggs was slightly elongated (Fig. 5b), consistent with the *P*(*r*) functions (Fig. 4c). The results also showed that the volume of the MP1 structure for the pasteurized frozen whole eggs was 2.8 times higher than that for the unpasteurized whole eggs (Table 2).

**Table 2:** Radius of gyration (*R*_g_), maximum distance (*D*_max_), and volume (*V*) data for the model structures.

Sample	MP1			MP2			MP3			Volume ratio (MP1/MP2)^1^	*I*(0) ratio/volume ratio (MP1/MP2)^2^
	*R*_g_ (Å)	*D*_max_ (Å)	*V* (A^3^)	*R*_g_ (Å)	*D*_max_ (Å)	*V* (A^3^)	*R*_g_ (Å)	*D*_max_ (Å)	*V* (A^3^)		
Whole egg	99.16	264.1	6.82 × 10^6^	25.25	83.59	8.30 × 10^4^	34.61	114.1	1.65 × 10^5^	82.2	0.42
PF whole egg	172.6	508.8	1.93 × 10^7^	24.97	80.02	8.25 × 10^4^	33.34	105.1	1.40 × 10^5^	233.7	0.17
Egg yolk	97.91	300.9	7.27 × 10^6^	–^3^	–	–	–	–	–	–	–
Egg white	–	–	–	25.39	90.71	8.24 × 10^4^	30.43	94.96	1.58 × 10^5^	–	–

^1^Ratio of the volume of the model structure for MP1 to that for MP2.

^2^The *I*(0) ratio (MP1/MP2) shown in Table 1 was divided by the volume ratio (MP1/MP2). This corresponds to the concentration ratio of the MP1 component relative to the MP2 component.

^3^Not detected.

### MP2 (ovalbumin)

The major component of MP2 was found to be ovalbumin (Fig. 2c). The scattering curves at the top of MP2 for unpasteurized whole eggs, unpasteurized egg white, and pasteurized whole eggs were in good agreement with that calculated from the crystal structure of ovalbumin (Supplementary Fig. 5a). The *R*_g_ values for these samples were 24–25 Å (Figs. 3c,d, 4d, Table 1). These values are in good agreement with the molecular size obtained for an ovalbumin monomer in a previous study (23.7 ± 0.4 Å)^31,34^, which suggests that ovalbumin monomers were in all the samples. Moreover, the Kratky plots and *P*(*r*) functions of these samples were almost the same (Fig. 4e,f). Thus, the structure of the MP2 component, mainly ovalbumin, was almost the same for the unpasteurized and pasteurized frozen whole eggs. There was a peak at *Q* =  ~ 0.07 Å^−1^ in the Kratky plot, indicating a globular structure (Fig. 4e). Additionally, the *P*(*r*) function had a peak at *r* =  ~ 28 Å and a *D*_max_ of ~ 80 Å (Fig. 4f), suggesting a slightly elongated structure. These results were consistent with the model structures of the MP2 component, which were not perfectly spherical but slightly elongated (Fig. 5d–f). Moreover, the model structures overlapped well with the crystal structure of ovalbumin (PDB ID: 1OVA)^35^ (Fig. 5d–f, Table 2), which indicates that the SEC-SAXS method can be used to obtain the overall structures of protein components in egg samples.

### MP 3 (ovotransferrin)

MP3 was found to mainly contain ovotransferrin (Fig. 2c). The scattering curves at the top of MP3 for unpasteurized whole eggs, unpasteurized egg white, and pasteurized whole eggs were in good agreement with that calculated from the crystal structure of ovotransferrin (Supplementary Fig. 5b). The Kratky plots indicate the globular structure of the MP3 component in all samples (Fig. 4h). The *R*_g_ values at the top of MP3 were 35 ± 2 Å, 31 ± 2 Å, and 32 ± 2 Å for unpasteurized whole eggs, unpasteurized egg white, and pasteurized frozen whole eggs, respectively (Fig. 4g, Table 1). Additionally, *D*_max_ was higher for whole egg samples than it was for unpasteurized egg white, which was consistent with the *R*_g_ values obtained (Fig. 4i). It has been reported that the *R*_g_ of purified ovotransferrin monomer is 30.4 Å^34^, which is in good agreement with the value obtained for the unpasteurized egg white. The results indicate higher *R*_g_ values for the whole egg samples than for the purified ovotransferrin monomer. This may be due to the coexistence of components in whole eggs that have molecular weights higher than that of ovotransferrin. One of such components is apovitellin 3 + 4 (110 kDa), which is an HDL from the granule fraction of egg yolk. Furthermore, the model structures were consistent with these results and overlapped with the crystal structure of ovotransferrin (PDB ID: 1OVT)^36^ (Fig. 5g–i).

### Comparison of model structures

After comparing the sizes of the model structures, it was found that the structure of MP1 (derived from the egg yolk plasma) was much larger than that of MP2 (ovalbumin) or MP3 (ovotransferrin). Additionally, the volume ratio of the MP1 structure to the MP2 structure was 82 for unpasteurized whole eggs and 234 for pasteurized frozen whole eggs (Table 2). On the other hand, the ratio of the *I*(0) value of MP1 to that of MP2 was 35 for unpasteurized whole eggs and 41 for pasteurized frozen whole eggs (Table 1). As previously indicated, *I*(0) is proportional to the product of the molecular weight and concentration of a protein. Therefore, the concentration ratio of the MP1 component to the MP2 component was calculated as (*I*(0) ratio)/(volume ratio), which was based on the assumption that the volume ratio corresponds to the molecular weight ratio. The concentration ratios thus obtained were 0.42 and 0.17 for the unpasteurized and pasteurized frozen whole eggs, respectively (Table 2). In both cases, the concentration of the MP1 component (mainly from egg yolk) was lower than that of the MP2 component (ovalbumin from egg white). This is consistent with the fact that the amount of egg white in a whole egg is higher than that of the yolk.

## Discussion

The results of the present study indicate that the differences between unpasteurized and pasteurized frozen whole eggs are mainly due to differences in the structures of proteins in egg yolk plasma eluted in MP1. The *R*_g_ value of MP1 for both unpasteurized whole eggs and egg yolk was ~ 100 Å, which is consistent with the average radius of proteins in plasma fraction^37,38^. In contrast, the MP1 of pasteurized frozen whole eggs (*R*_g_ ~ 180 Å) eluted earlier than the MP1 of any unpasteurized sample did, which indicated that large aggregates had been formed. It should be noted that since it was not possible to measure *R*_g_ above ~ 270 Å in the present measurement, we cannot rule out the presence of the components larger than this value. Thus, the *R*_g_ obtained here for MP1 may correspond to the lower limit of the molecular sizes of the components present in this fraction.

Furthermore, we found that apovitellenins I and II form aggregates that are larger than 0.45 μm, which are removed during centrifugation and filtration before a sample is subjected to HPLC. It has been previously indicated that freezing and thawing of egg yolk results in the aggregation of plasma-derived proteins^39–44^. The present results are consistent with the previous results and further show that plasma-derived proteins form large aggregates upon freezing and thawing, even in whole eggs. Taken together, our results suggest that proteins in egg yolk plasma, especially apovitellenins I and II, are sensitive to temperature changes that occur as a result of heating during pasteurization, cooling during freezing, and reheating during thawing. Consequently, these proteins are prone to aggregation under different temperature conditions.

Apovitellenin II is a glycoprotein that is readily soluble in salt solutions^45^. The SDS-PAGE results showed that the amount of apovitellenin II in unpasteurized whole eggs was lower than that in unpasteurized egg yolk (Fig. 2). This suggests that salts in egg white affect the structures of lipoproteins in egg yolk plasma. In a previous study, it was found that apovitellenin I forms aggregates under freezing conditions^46^. However, to our knowledge, there are few reports on the aggregation of apovitellenin II. We believe that further studies must be conducted on the structural properties of apovitellenin II to develop pasteurized frozen whole eggs that are similar to unpasteurized ones.

In this study, we investigated whether SEC-UV and SEC-SAXS can be used to effectively compare the structural characteristics of unpasteurized and pasteurized frozen whole eggs. The SEC-UV technique was used to study differences between the egg samples. Additionally, the assignment of eluted proteins was done by using SEC-UV and SDS-PAGE in combination. We used SEC-SAXS, which is a new technique that has recently received much attention, to elucidate the molecular sizes and shapes of multiple components in the egg samples. The results showed clear differences in the overall structures of egg yolk-derived proteins between unpasteurized and pasteurized frozen whole eggs. This indicates that both SEC-UV and SEC-SAXS can be used to successfully compare the structural characteristics of unpasteurized and pasteurized whole eggs. SEC-UV can be easily performed in a laboratory; however, SAXS may require a large facility. Therefore, it is recommended to perform SEC-UV first, followed by SEC-SAXS for further investigation to compare the physical properties of raw and processed eggs. Generally, HPLC experiments are performed on the samples that are centrifuged and filtered before the analysis and are passed through a narrow channel. Therefore, other methods such as microscopy and ultracentrifugation are required for the analysis of highly viscous and aggregation-prone macromolecules such as the ovomucin network.

In conclusion, unpasteurized and pasteurized frozen whole hen eggs were successfully compared using SEC-UV and SEC-SAXS methods in the present study. The differences between the eggs were mainly attributed to aggregated components in the egg yolk-derived plasma fraction. This suggests that the structures of the proteins in egg yolk plasma, especially apovitellenins I and II, are sensitive to temperature changes, such as heating during pasteurization, cooling during freezing, and reheating during thawing. Therefore, the next challenge will be to develop a technology to process whole eggs that can be stored for long periods without affecting the structure of egg yolk plasma. We believe that the SEC-UV and SEC-SAXS techniques will be useful for comparing the characteristics of processed eggs to those of raw eggs in future studies. Furthermore, they may be useful in determining molecular sizes and shapes of multiple components in various complex biological systems such as whole eggs.

## Methods

### Sample preparation

In this study, unfertilized hen eggs taken from domestic fowls, *Gallus gallus domesticus*, were used. The fresh shell eggs were purchased from a local chicken farm. Unpasteurized egg yolks and egg whites were prepared by removing the shells of raw eggs and separating the yolks from the egg whites. The separated components were then homogenized and stirred gently. Unpasteurized whole eggs were prepared by gentle homogenization without separation of the yolks and egg whites. The separation and homogenization were performed at room temperature. Pasteurized frozen whole eggs were prepared by placing one kilogram of the homogenized whole eggs in a polyethylene bag, heating it at 60 °C for 3.5 min, and then rapidly freezing it at − 30 °C using a blast chiller (AL-14MC, FMI Corporation, Tokyo, Japan) over 2 h and were stored in the chiller for three months. Pasteurized frozen whole eggs were thawed by leaving them in a refrigerator at 4 °C overnight before being brought to ~ 25 °C. To prevent spoilage, 0.02% sodium azide was added to the unpasteurized samples.

The egg samples, especially the pasteurized frozen whole eggs, were very viscous; therefore, all the samples were diluted 25-fold with the elution buffer (50 mM sodium phosphate [pH 7.8] and 550 mM NaCl) before they were subjected to the SEC analysis to prevent clogging of the HPLC flow path. Previous studies have used the phosphate buffer for egg white and whole hen eggs and reported that hen egg proteins are well dissolved in phosphate buffer^47,48^. Prior to the HPLC analysis, each sample solution was centrifuged at 20,000×*g* for 30 min at 4 °C to remove precipitates. After centrifugation, the supernatant was filtered through a membrane filter unit (Millipore Sigma, Burlington, MA, USA) with a pore size of 0.45 μm.

### SEC-UV analysis

Twenty microliters of sample solution prepared as described above was subjected to gel filtration through a Superdex 200 Increase 3.2/300 column (column volume, 2.4 mL; Cytiva, Marlborough, MA, USA) attached to an HPLC system (LP-20AP; Shimadzu, Kyoto, Japan). The flow rate was 0.1 mL/min for the first 7 min before the void volume, and 0.05 mL/min after 7 min. The elution process was monitored by UV absorption at 280 nm. All SEC-UV analyses were performed more than twice to assess the reproducibility of the method.

The eluate from the column was collected in 100 µL fractions. SDS-PAGE was performed using a 5–20% gradient gel (ATTO, Tokyo, Japan) to determine the components present in each fraction. All samples were treated with 5% of 2-mercaptoethanol to reduce disulfide bonds and heated to 100 °C for 5 min before they were applied on the gel.

### SEC-SAXS analysis

The SEC-SAXS measurements were performed at the beamline BL-10C of the Photon Factory at the High Energy Accelerator Research Organization (KEK), Tsukuba, Japan. The same column (Superdex 200 Increase 3.2/300) that was used in the SEC-UV analysis was connected to another HPLC system (Waters Co., Milford, MA, USA) installed in the beamline. The flow rate was 0.05 mL/min. The sample solution flowing through a quartz-windowed flow path (path length, 1 mm) was irradiated with a monochromatic X-ray beam (1.488 Å). The temperature of the flow path was maintained at 25 °C in a thermostat circulating water bath. Scattering images were acquired using a PILATUS 2 M detector (DECTRIS Ltd., Baden, Switzerland). Data were collected in the range of a scattering vector *Q* from 0.0048 to 0.26 Å^−1^ (*Q* = 4π sin(*θ*/*λ*), *λ* is the wavelength, and 2*θ* is the scattering angle). Each scattering curve was measured after irradiation with X-rays for 10 s. The number of scattering curves obtained from one SEC-SAXS analysis was 390. The integrated scattering intensity was obtained by summing the scattering intensities at all the scattering angles. All measurements were performed in duplicate to assess reproducibility.

### SAXS analysis

The structures at the three major peaks in the elution profiles (MP1, MP2, and MP3) were analyzed in detail as follows. First, the average of the scattering before (0–8.5 min) and/or after (56.8–65 min) the elution of an egg sample was used as the scattering by the buffer, which was then subtracted from the scattering by an egg sample. To increase the signal-to-noise ratio of the scattering curve, several data points were binned together. *R*_g_ and *I*(0) were then calculated according to the Guinier approximation by fitting the following equation into the region where *R*_g_∙*Q* < 1.3 holds in the Guinier plot^49^:

1 $${\text{ln}}\;I\left( Q \right) \, = {\text{ ln}}\;I\left( 0 \right) \, {-}R_{{\text{g}}}^{{2}} Q^{{2}} /{3}{\text{.}}$$

SAngler^50^ and KaleidaGraph (Synergy Software, Reading, PA, USA) softwares were used for fitting. The *R*_g_ values around the peaks (MP1, MP2, and MP3) were found to be almost constant (Fig. 3c,d) and the shapes of the scattering curves remained unchanged. However, the *I*(0) values changed because of changes in solute concentration, indicating that there was no interparticle interference effect.

The pairwise distance distribution function *P*(*r*) was calculated using the GNOM software included in the ATSAS software package^51^. Modeling of the overall structures was performed using DAMMIF software^32^. During DAMMIF modeling, 10 modeling runs with DAMMIN software were performed and the average structures were obtained using DAMSEL, DAMSUP, and DAMAVER softwares^32^. Finally, DAMMIN modeling was performed using the average structure as the initial structure to obtain the final model structure. The range of scattering curves used in the modeling was determined based on the values of Los and Scale, which indicate the accuracy of the modeling. Structural modeling was also performed using GASBOR software^33^. The normalized spatial discrepancy value, which is a parameter that indicates the similarity between the DAMMIF and GASBOR model structures, was calculated using the SUPCOMB program^52^. The range of the scattering curves used in the GASBOR model was determined based on this value. GASBOR modeling was performed 10 times, and the best model structure was selected based on the χ^2^ value, which represents the modeling accuracy. The model structures obtained using DAMMIF and GASBOR were superimposed and drawn using Chimera software version 1.14 (Resource for Biocomputing, Visualization, and Informatics, University of California, San Francisco, San Francisco, CA, USA)^53^. The structure of the MP1 component could not be modeled with GASBOR because of the high molecular weight of the component.

The calculation of scattering curves from the crystal structures of ovalbumin (PDB ID: 1OVA) and ovotransferrin (PDB ID: 1OVT) were performed using the CRYSOL software included in the ATSAS package^51^.

## Supplementary Information

Supplementary Information.

## Data Availability

The authors declare that all data supporting the findings of this study are available within the paper and its Supplementary Information file.
