# Supplementary material for: A comparative study of unpasteurized and pasteurized frozen whole hen eggs using size-exclusion chromatography and small-angle X-ray scattering
Source: Sci Rep. 2022 Jun 2;12:9218. doi: 10.1038/s41598-022-12885-z (PMC9163139; doi:10.1038/s41598-022-12885-z)
Supplement: Supplementary file 1 — Supplementary Information. [file 41598_2022_12885_MOESM1_ESM.pdf]

Supplementary Information

A comparative study of unpasteurized and pasteurized frozen whole hen eggs using size-exclusion chromatography and small-angle X-ray scattering

Yoshiki Oka[1], Hiroko Yukawa[2], Hisashi Kudo[1,6], Koji Ooka[3,7], Manami Wada[1], Shunji Suetaka[1], Mari Chang[3], Hidenobu Kawai[1], Ryouji Tanaka[2], Masahiro Ichikawa[2], Takahisa Suzuki[2], Yuuki Hayashi[1,4], Akihiro Handa[2,5], and Munehito Arai[1,3,*]

[1] Department of Life Sciences, Graduate School of Arts and Sciences, The University of Tokyo, 3-8-1 Komaba, Meguro, Tokyo 153-8902, Japan

[2] Institute of Technology Solution, R&D Division, Kewpie Corporation, Sengawa Kewport, 2-5-7 Sengawa, Chofu, Tokyo 182-0002, Japan

[3] Department of Physics, Graduate School of Science, The University of Tokyo, 3-8-1 Komaba, Meguro, Tokyo 153-8902, Japan

[4] Environmental Science Center, The University of Tokyo, 7-3-1 Hongo, Bunkyo, Tokyo 113-0033, Japan

[5] Division of Life Science, School of Science and Engineering, Tokyo Denki University, Ishizaka, Hatoyama-machi, Hiki-gun, Saitama 350-0394, Japan

[6] Present address: Graduate School of Science, Technology and Innovation, Kobe University, 1-1 Rokkodai-cho, Nada, Kobe 657-8501, Japan

[7] Present address: Komaba Organization for Educational Excellence, College of Arts and Sciences, The University of Tokyo, 3-8-1 Komaba, Meguro, Tokyo 153-8902, Japan

*Corresponding author:
Munehito Arai, Ph.D.
Department of Life Sciences, Graduate School of Arts and Sciences, The University of Tokyo, 3-8-1 Komaba, Meguro, Tokyo 153-8902, Japan
Phone: +81-3-5454-6751
E-mail: arai@bio.c.u-tokyo.ac.jp

Supplementary Table 1. Molecular weights of egg proteins in egg yolk[8,21]

Molecular weight (kDa)	Protein	Plasma or granule fraction
203	apovitellenin VI	plasma (LDL)
203	γ-livetin	plasma
122	apovitellenin Va	plasma (LDL)
110	apovitellin 3+4	granule (HDL)
85	apovitellenin V	plasma (LDL)
78	apovitellin 5+6	granule (HDL)
68	apovitellenin IV	plasma (LDL)
47	apovitellin 7	granule (HDL)
34–36	β-livetin	plasma
20	apovitellenin II	plasma (VLDL)
17	apovitellenin I (dimer)	plasma (VLDL)
9	apovitellenin I (monomer)	plasma (VLDL)
5	apolipoprotein CII	plasma (VLDL)

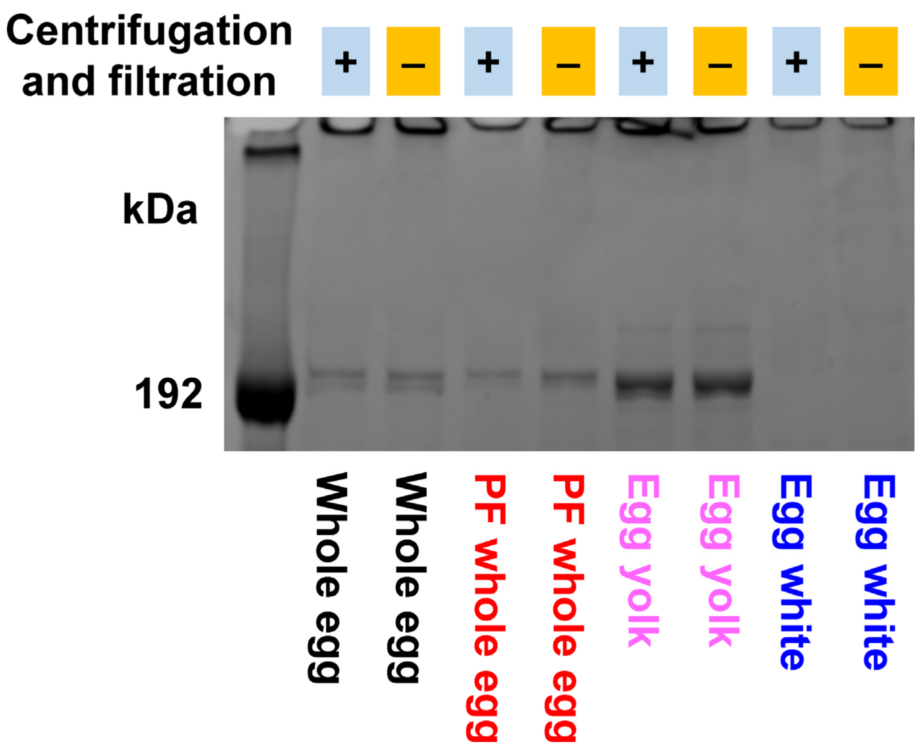

Supplementary Figure 1. SDS-PAGE analysis of egg samples before (–) and after (+) centrifugation and filtration. Only the bands for samples with high molecular weights are shown. Large aggregates were formed in the unpasteurized and pasteurized frozen (PF) whole eggs and were removed by centrifugation and filtration of the samples. This resulted in thinner bands after the treatment. The decrease in band intensity was particularly noticeable for PF whole eggs. For unpasteurized egg white, bands above 200 kDa were not clearly detected.

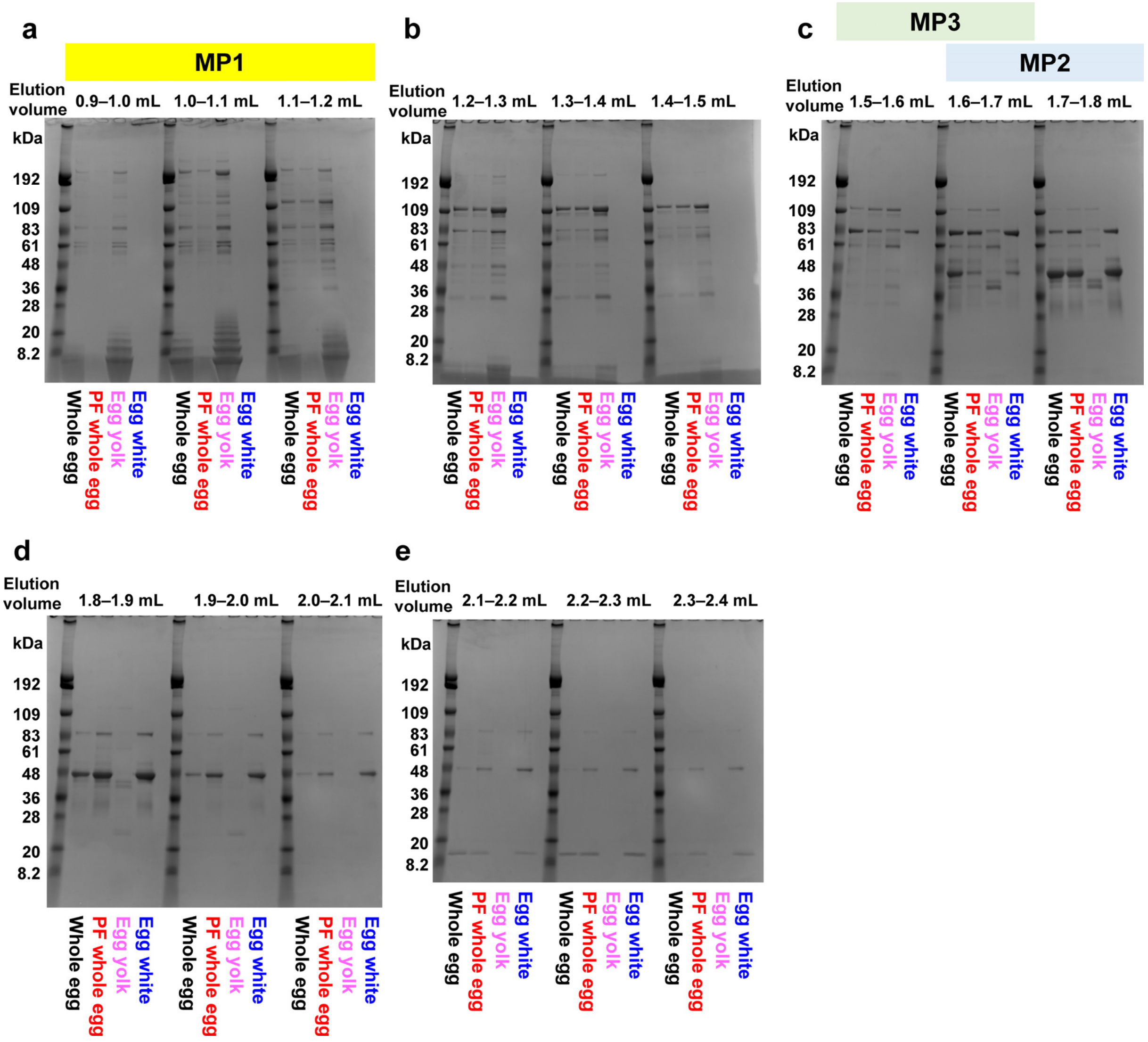

Supplementary Figure 2. SDS-PAGE analysis of SEC elution fractions. For each fraction, lanes for molecular weight markers, unpasteurized whole eggs, pasteurized frozen (PF) whole eggs, unpasteurized egg yolk, and unpasteurized egg white are shown. (a) Fractions corresponding to MP1 (elution volume of 0.9–1.2 mL). (b) Fractions for the elution volume of 1.2–1.5 mL. (c) Fractions corresponding to MP2 and MP3 (elution volume of 1.5–1.8 mL). (d) Fractions for the elution volume of 1.8–2.1 mL. (e) Fractions for the elution volume of 2.1–2.4 mL. Panels (a)–(c) are the same as those of Fig. 2.

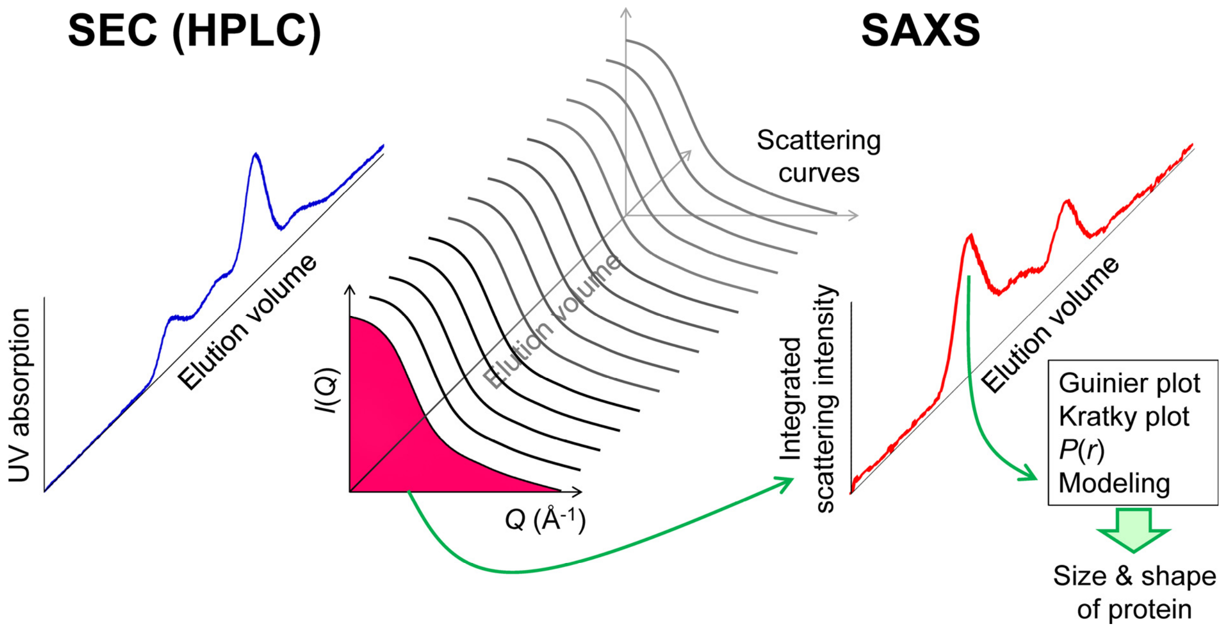

Supplementary Figure 3. Schematic of SEC-SAXS technique. (Left image) SEC is performed via HPLC. An example of the elution profile monitored by UV absorption is shown. (Center image) The SEC eluate is continuously irradiated with X-rays. A scattering curve is obtained by irradiating a sample with X-rays for 10 s. The integrated scattering intensity is calculated by summing the scattering intensities at all angles of the scattering curve, and the elution profile is monitored using the integrated scattering intensity obtained (right image). The molecular size and shape of each protein in the eluate are then determined by analyzing the Guinier and Kratky plots of the scattering curve, calculating the *P*(r) function, and modeling the overall structure.

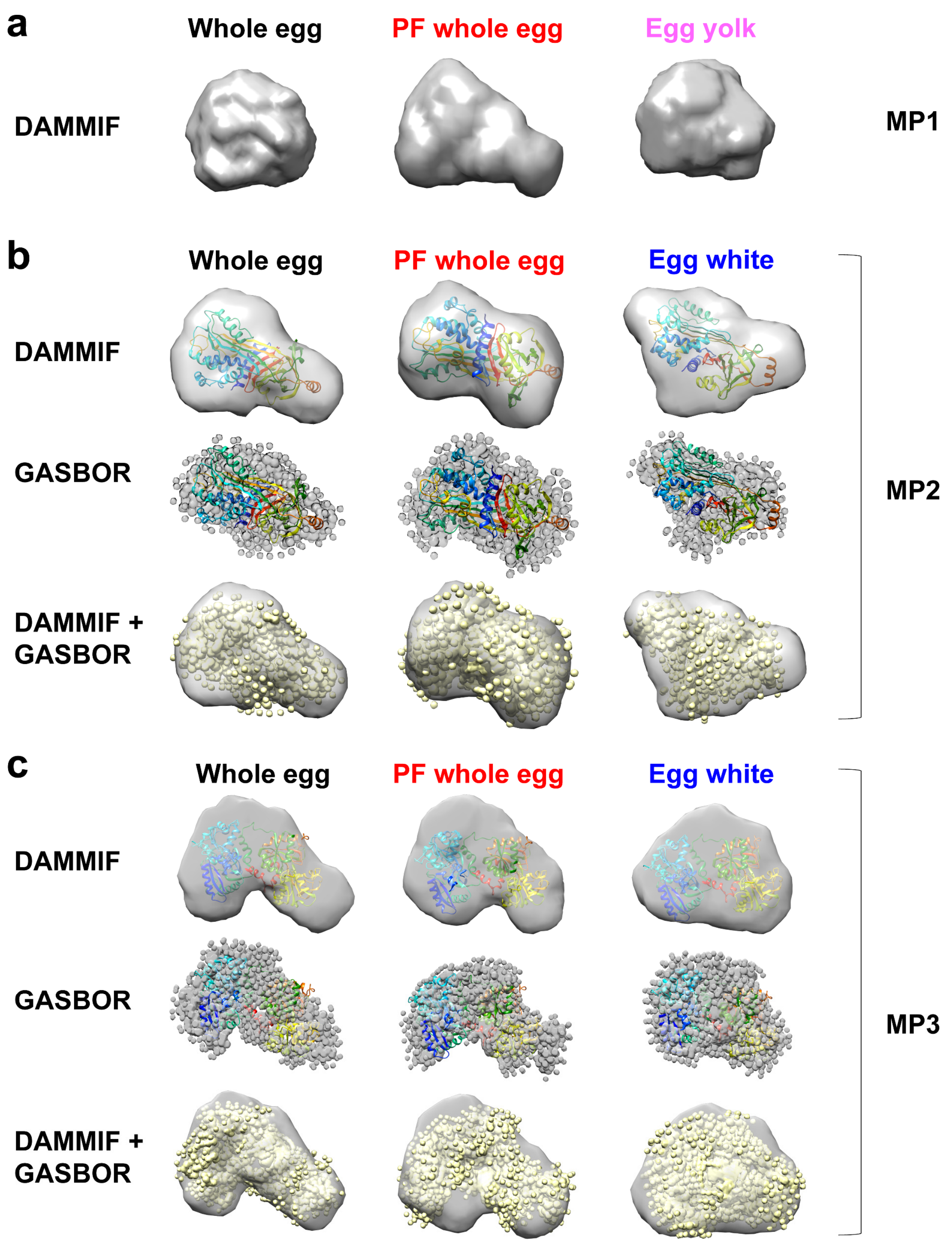

Supplementary Figure 4. Structural modeling of egg samples using DAMMIF and GASBOR softwares. The structures modeled by DAMMIF are the same as those shown in Fig. 5. Model structures at the tops of (a) MP1, (b) MP2, and (c) MP3 for unpasteurized whole eggs (left), pasteurized frozen (PF) whole eggs (middle), and unpasteurized egg yolk or egg white (right). For MP2 and MP3, the structures modeled by GASBOR are superimposed on the structures modeled by DAMMIF. The crystal structures of ovalbumin and ovotransferrin are superimposed onto the model structures of MP2 and MP3, respectively. The figures were drawn using Chimera software version 1.14 (Resource for Biocomputing, Visualization, and Informatics, University of California, San Francisco, San Francisco, CA, USA; https://www.cgl.ucsf.edu/chimera/)[53].

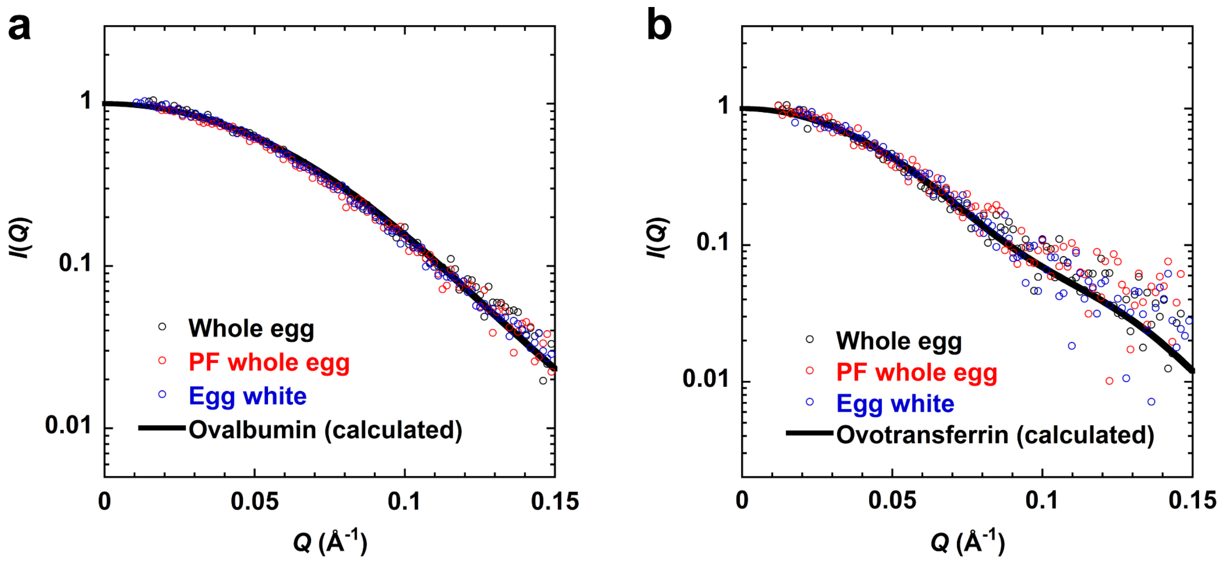

Supplementary Figure 5. Comparison of the experimental and theoretical scattering curves. The experimentally observed scattering curves of MP2 (a) and MP3 (b) for unpasteurized whole eggs, pasteurized frozen (PF) whole eggs, and egg white, which are normalized by the respective *I*(0) values, are overlaid with the theoretical scattering curve of ovalbumin (PDB ID: 1OVA) (a) and ovotransferrin (PDB ID: 1OVT) (b) calculated by the CRYSOL software[51]. The figures were created with KaleidaGraph 4.1.0 (Synergy Software, Reading, PA, USA; https://www.synergy.com/).

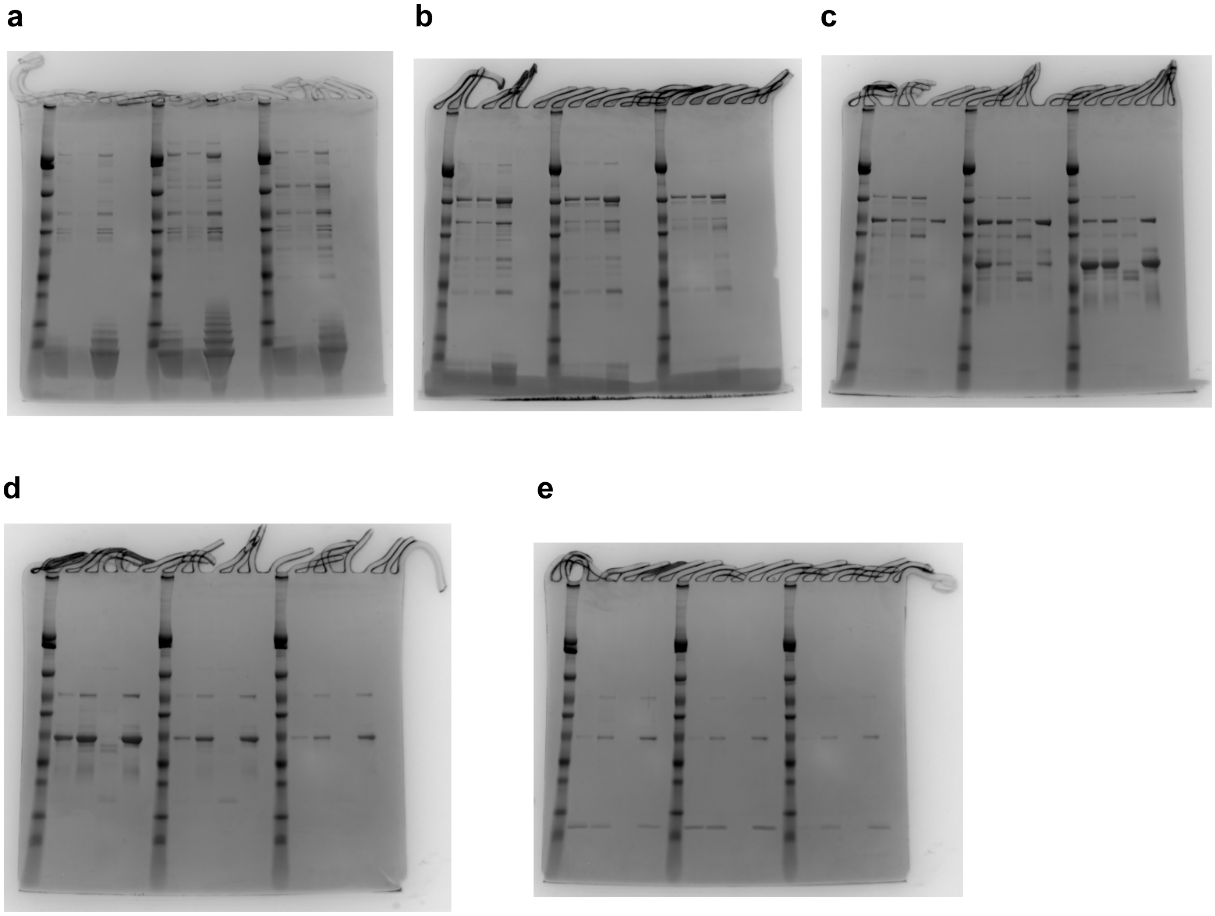

Supplementary Figure 6. The uncropped gel images of Fig. 2 and Supplementary Figure 2. Panels (a)–(c) correspond to those of Fig. 2. Panels (a)–(e) correspond to those of Supplementary Figure 2.

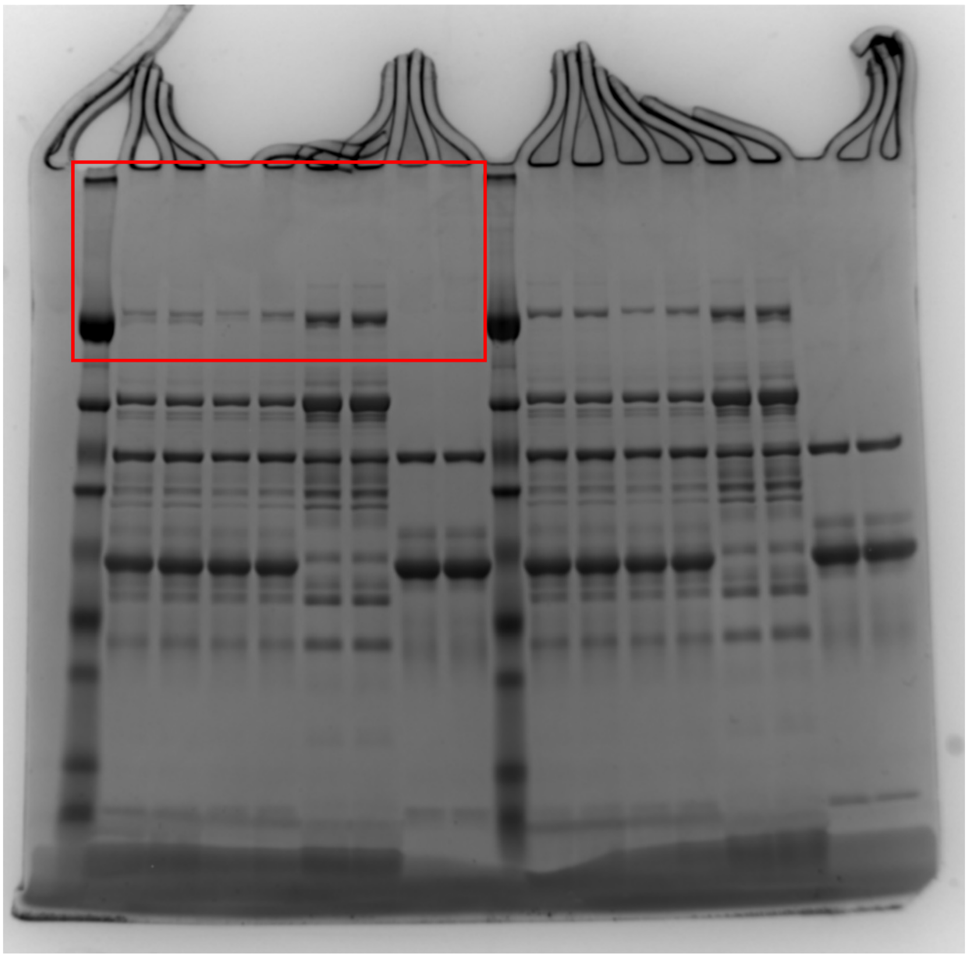

Supplementary Figure 7. The uncropped gel image of Supplementary Figure 1. The red box indicates the cropped region.
